# COVID-19 mitigates the response to TKIs in patients with CML via the inhibition of T-cell immunity

**DOI:** 10.3389/fimmu.2024.1452035

**Published:** 2024-11-20

**Authors:** Na He, Guosheng Li, Jinting Liu, Wancheng Liu, Ruifeng Tian, Daoxin Ma

**Affiliations:** Department of Hematology, Qilu Hospital of Shandong University, Jinan, China

**Keywords:** Chronic myeloid leukemia, COVID-19, BCR-ABL P210, T cell immunity, TKIs

## Abstract

**Introduction:**

Chronic myeloid leukemia (CML) is a severe hematological malignancy characterized by BCR-ABL fusion gene. The advent of tyrosine kinase inhibitors (TKIs) targeting BCR-ABL has improved the landscape of CML treatment dramatically. The occurrence of coronavirus disease 2019 (COVID-19) has challenged many cancers. However, its effect on TKI therapy of CML remains unknown.

**Methods:**

In this study, we collected peripheral blood from chronic phase CML patients treated with TKIs at low-level BCR-ABL P210 during COVID-19 pandemic, and determined the alterations of BCR-ABL P210 by applying the well-established BCR-ABL P210 detection system.

**Results:**

Our results showed that the level of BCR-ABL P210 of CML patients was significantly elevated shortly after contracting COVID-19, and then recovered to pre-infection level within one month. The elevated degree of P210 was positively correlated with the duration of COVID-19. And the level of P210 was elevated in CML patients that took COVID-19 vaccination. Furthermore, lymphocyte subsets and cytokine detections were performed by flow cytometry to analyze the alteration of immune responses. Our results showed that effector CD8+ T (Teff) cells were significantly downregulated while naïve CD8+ T cells or Treg cells were obviously upregulated in P210-elevated CML patients after contracting COVID-19 compared to that in P210-unchanged or decreased CML patients. Moreover, the SARS-CoV-2 pseudovirus was constructed to further determine its effects. The results showed that the level of BCR-ABL P210 was upregulated upon transfection of SARS-CoV-2 pseudovirus into blood samples of CML patients.

**Discussion:**

Our results demonstrate that COVID-19 suppresses the immune activity and consequentially elevates the level of BCR-ABL P210 of CML patients.

## 1 Introduction

Chronic myeloid leukemia (CML) is a severe and well-studied hematological malignancy characterized by clonal myeloproliferative abnormalities. Abnormally activated tyrosine kinases play important roles in the development of CML through the activation of various downstream signaling pathways, leading to an imbalance between cell survival and apoptosis, abnormal cell transformation, and exceptional self-renewal capacity, thereby promoting leukemogenesis (1). The hallmark of CML is the Philadelphia (Ph) chromosome, which is derived from the t (9;22) (q34; q11) translocation and results in the fusion of the BCR and ABL genes. The variation in the breakpoint within the BCR partner produces different hybrid genes encoding varying BCR-ABL isoforms (2). The most common variant is P210^BCR-ABL^, which is expressed in most CML patients and presents with strong tyrosine kinase activity (3). The development of tyrosine kinase-related drugs has drastically changed the natural history of CML. Tyrosine kinase inhibitors (TKIs), such as imatinib, dasatinib, and bosutinib, are extensively employed as targeted therapeutic strategies to inhibit tyrosine kinases in CML patients. With the use of TKIs for CMI treatment, survival and remission rates have greatly increased; moreover, BCR-ABL P210 level plays an important role in minimal residual disease (MRD) detection and is an indicator of the withdrawal of TKIs (4).

COVID-19 has emerged and rapidly swept the globe, causing enormous human health challenges, especially for patients with cancer. Patients with cancer are more susceptible to COVID-19 and have worse outcomes from COVID-19 than those without cancer (5). However, patients with CML, a long-term chronic disorder maintained by regular TKI treatment, have no significantly increased risk of COVID-19 (6, 7). Moreover, compared with other hematological malignancies, the mortality rate of CML patients is lower, and most CML patients are asymptomatic (8, 9). Interestingly, studies have shown that CML patients may still have an increased risk of complications after contracting COVID-19 (10). As BCR-ABL P210 plays an important role in the prognosis and follow-up of TKI-treated CML patients, investigating whether COVID-19 could cause dynamic changes in BCR-ABL P210 and further influence the effect of TKI therapy is highly important.

COVID-19 has been reported to activate humoral and cellular immune responses (11). For COVID-19 in CML patients, immune responses are also initiated after infection, and the duration of natural anti-SARS-CoV-2 antibodies is 8–12 months, similar to that in the general population (12). In addition, some studies have demonstrated that TKIs play antiviral and protective roles in CML patients after contracting COVID-19 (13), whereas other studies have shown that TKI treatment does not affect the risk or severity of COVID-19 (12). Therefore, the potential impact of COVID-19-induced immune system disorders on the efficacy of TKI treatment in CML patients remains insufficiently investigated.

## 2 Materials and methods

### 2.1 Patient samples

Peripheral blood samples were collected from healthy volunteers and chronic phase CML patients treated with TKIs in Qilu Hospital of Shandong University from October 2022 to January 2024. All CML patients included in this study were followed consecutively in our institute. The informed consent was obtained from all patients in accordance with the Declaration of Helsinki. The study was approved by the Medical Ethical Committee of Qilu Hospital of Shandong University.

### 2.2 Establishment of internal quality control of BCR-ABL P210 detection

#### 2.2.1 Levey-Jennings (L-J) quality control chart

We prepared 3 kinds of internal quality control (IQC) samples, in which high IQC sample was P210-positive K562 cells, medium or low IQC sample was K562 cells 1000-fold or 3000-fold diluted with P210-negative Jurkat cells. We determined BCR-ABL P210 expression of these 3 IQC samples for twice a week consecutive for 18 times. After acquiring the results, we calculated the mean value ( $\bar{\text{x}}$ ) and standard error (s). Then we draw the Levey-Jennings (L-J) quality control chart using $\bar{\text{x}}$ as the center line, $\bar{\text{x}}$ ± 1s as the control line, $\bar{\text{x}}$ ± 2s as the warning line, and $\bar{\text{x}}$ ± 3s as the out-of-control line.

#### 2.2.2 Westgard multi-rule quality control judgment

L-J quality control chart is judged by Westgard multi-rule standards (14). In brief, 1_3s_ out-of-control rule is the detective value exceeding $\bar{\text{x}}$ ± 3s, 1_2s_ warning rule is the detective value between $\bar{\text{x}}$ ± 2s to $\bar{\text{x}}$ ± 3s, 2_2s_ out-of-control rule is two detective levels exceeding the $\bar{\text{x}}$ ± 2s simultaneously or sequentially, R4s out-of-control rule is the difference value of two quality control substances exceeding 4s, and 4_1s_ out-of-control rule is the detective value exceeding the $\bar{\text{x}}$ ± 1s control line in the same direction for 4 consecutive times. We applied these rules to estimate the accuracy and stability of BCR-ABL P210 results.

### 2.3 BCR-ABL P210 detection

#### 2.3.1 BCR-ABL P210 detection by real-time PCR

PBMCs were isolated from peripheral blood of CML patients. Total RNA was extracted by using Trizol reagent (Invitrogen, USA). A total of 1 μg RNA was used to detect the expression of BCR-ABL P210 by applying one-step RT-qPCR kit (Shanghai Righton gene Biotechnology Co., Ltd, Shanghai, China). The real-time PCR contained, in a final volume of 15μL, 4.8μL of BCR-ABL-210 PCR reaction solution, 1.2μL of mixed enzyme solution, and 9μL of total RNA. The quantitative PCR was performed on the ABI PRISM 7500 real-time PCR instrument (PE Applied Biosystems, Foster City, CA, USA) in accordance with the manufacturer’s instruction. The PCR products were analyzed by standard curve analysis and the results were expressed as the ratio of BCR-ABL P210 copy number to the ABL copy number.

#### 2.3.2 BCR-ABL P210 detection by digital PCR

A total of 500ng RNA was used to detect the expression of BCR-ABL P210 by applying one-step RT-dPCR kit (Suzhou Sniper Medical Technologies Co.,Ltd, Suzhou,China). The digital PCR contained, in a final volume of 22 μL, 11 μL of BCR-ABL-210 PCR reaction solution, 2 μL of mixed enzyme solution, and 8 μL of total RNA. The digital PCR was performed on the Sniper Digital PCR All-in-One System (DQ24-Dx, Suzhou Sniper Medical Technologies Co.,Ltd, Suzhou,China) in accordance with the manufacturer’s instruction. The Digital PCR System divided the reaction system into about 28,000 droplets and each droplet can independently complete the PCR amplification reaction. By detecting the fluorescence signal of each droplet, the copy number of BCR-ABL P210 or ABL in the whole reaction system was calculated according to the Poisson distribution.

### 2.4 Absolute counting of lymphocyte subsets

A total of 50μL peripheral blood of CML patients was transferred into BD Trucount absolute counting tube. Then, 20μL lymphocyte subset detection reagent containing flow cytometry-6 colors antibodies against CD3 FITC, CD16/CD56 PE, CD45 PerCP cy5.5, CD4 PC7, CD19 APC, CD8 APC-cy7 was added to the bottom of the test tube, and incubated at room temperature for 20 minutes. Finally, after being added 450μL BD hemolysin and incubated at room temperature for 10 minutes, the cells were detected immediately using a Gallios flow cytometry (Beckman Coulter, CA, USA).

### 2.5 Relative counting of T cell subsets

The peripheral blood of CML patients was lysed by using erythrocyte lysate (BD biosciences company, USA), and centrifuged at 350g for 5 minutes to remove erythrocytes. After being re-suspended in PBS, the cells were transferred into 2 test tubes, with 1×10^6^ cells/100μL in each tube. Then the cells were added with fluorescence-labelled antibodies against CD45 KO, CD3 APC, CD4 PE, CD8 APC-H7, CD45RA PC7 and CD62L PerCP cy5.5 in one tube; and added with fluorescence-labelled antibodies against CD45 KO, CD3 FITC, CD4 PerCP cy5.5, CD25 APC, CD127 PE, CD45RA PC7and HLA-DR APC-H7 in the other tube. After being incubated at room temperature for 20 minutes and washed with PBS, the cells were detected immediately using a Gallios flow cytometry (Beckman Coulter, CA, USA).

### 2.6 Cytokine detection

Cytokine levels were detected by using multiplexed microsphere-based flow cytometric assays in accordance with the manufacturer’s instruction (Biolegend, USA). The samples were detected by using flow cytometry. The concentrations were calculated from a standard curve according to the manufacturer’s protocol.

### 2.7 Infection of SARS-CoV-2 pseudovirus

The plasmids of PNL’-Luc, pcDNA3.1 and nCOV-S were supported by the Suzhou Institute of System Medicine. After packaging the SARS-CoV-2 pseudovirus, we infected the PBMCs of CML patients with SARS-CoV-2 pseudovirus at the MOI of 1:10. Then we determined the expression level of BCR-ABL P210.

### 2.8 Statistical analysis

Statistical analysis was performed using GraphPad Prism 8 software. T test was used to compare the BCR-ABL P210 transcript. Chi-square test was used to analyze changes in the two sequential samples and the association between P210 level alteration and the interval from COVID-19 onset to detection. Difference was meaningful at *p* < 0.05.

## 3 Results

### 3.1 Characteristics of the subjects

The demographics and disease characteristics of the CML patients are presented in **Table 1**. During the outbreak peak of COVID-19, a total of 109 chronic-phase CML patients treated with TKIs were enrolled in this study; 60 (55%) were male, 49 (45%) were female, and the median age was 44 (range from 11 to 81) years. Among them, 53 individuals were affected by COVID-19, with a median age of 37 years, whereas 56 patients were not affected, with a median age of 49 years. For COVID-19-positive CML patients, 3 individuals were CCyR (complete cytogenetic response, CCyR), 28 individuals were MMR (major molecular response, MMR), 22 individuals were MR4.5 (molecular response, MR4.5). Most presented with symptoms such as fever (98%), respiratory symptoms such as rhinobyon, rhinorrhea, tachypnea, dyspnea, cough, sputum and pharyngalgia (77%), and other symptoms such as arthralgia, headache, and sleepiness (67.9%). Moreover, a few patients presented digestive disorders such as emesis and diarrhea (18.9%). For COVID-19-negative CML patients, 4 individuals were CCyR, 27 individuals were MMR, and 25 individuals were MR4.5. As for the molecular effect of COVID-19 on CML clinical endpoints, we found that after contracting COVID-19, 5 (9.4%) patients transfered from MR4.5 to MMR, 1(1.9%) patient transfered from MMR to CCyR, 1(1.9%) patient transfered from MR4.5 to CCyR, 2 (3.8%) patients transfered from MMR to MR4.5, and 44 (83%) patients have no change. These data indicate that 13.2% of CML patients lose their deep molecular response after contracting COVID-19.

**Table 1:** Patient characteristics (n = 109).

Variable		COVID-19	Non-COVID-19
Sex	male	32	28
	female	21	28
Age	median	37	49
	≥60 years	5	18
Treatment outcome	CCyR	3	4
	MMR	28	27
	MR4.5	22	25
	fever	98	NA
Comorbidity (%)	respiratory infection	77	NA
	digestive disorders	18.9	NA
	other symptoms	67.9	NA
	No	NA	NA
Interval since diagnosis COVID-19, days, median (range)		27 (7-49)	NA
Baseline BCR-ABL 1^IS^ transcript (%), median (range)		0.0083 (0-0.28)	0.0044 (0-0.18)

COVID-19, Coronavirus disease 2019; CCyR, Complete cytogenetic response; MMR, Major molecular response; MR4.5, Molecular response 4.5; IS, International Scale; NA, No available.

### 3.2 Establishment of the BCR-ABL P210 IQC system

**Figure 1:**
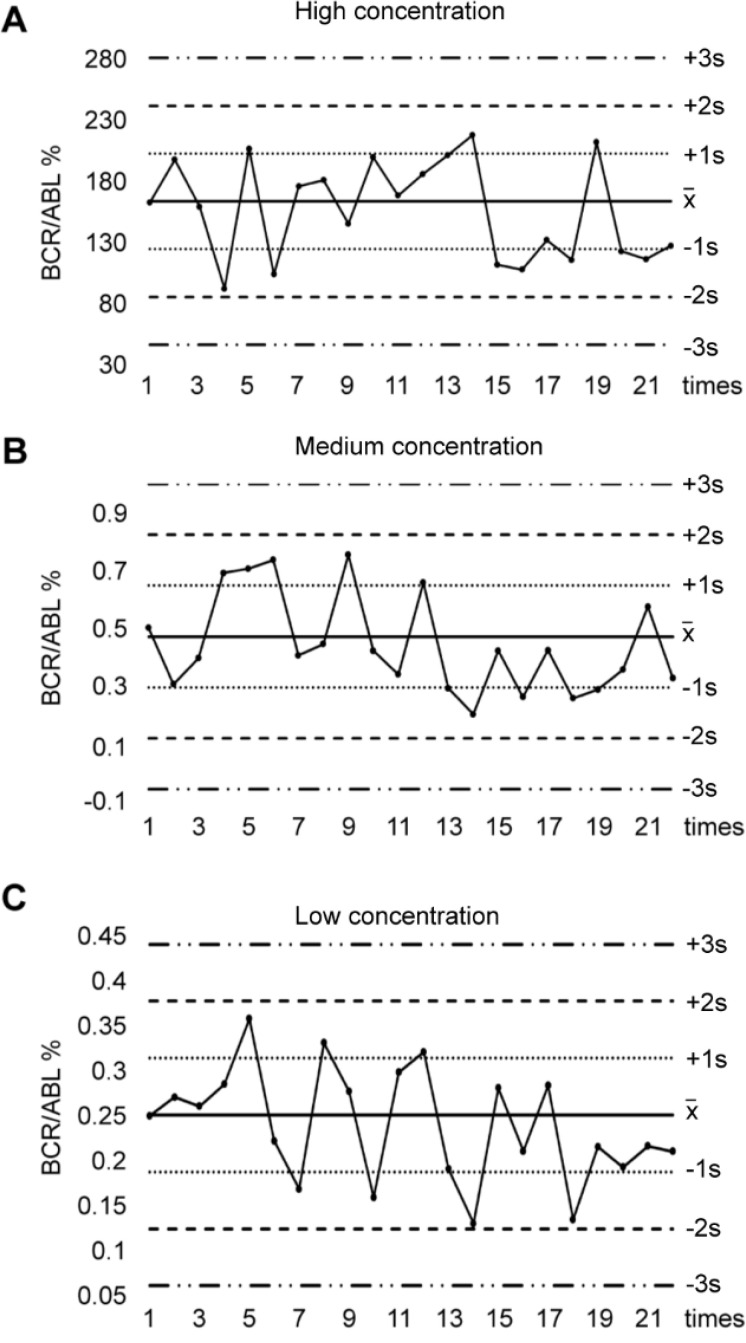
Levey - Jennings control chart for IQC in BCR-ABL P210 detection. **(A)** BCR-ABL P210 quality control results of high concentration of IQC samples. **(B)** BCR-ABL P210 quality control results of medium concentration of IQC samples. **(C)** BCR-ABL P210 quality control results of low concentration of IQC samples.

The detection of BCR-ABL P210 expression at the MRD level was unstable and fluctuated. To ensure the reliability of BCR-ABL P210 detection, we first established a BCR-ABL P210 IQC system. We evaluated the IQC samples 18 times within 2 months to monitor the methodological stability of BCR-ABL P210 detection. Our results revealed that the BCR-ABL P210 quality control results of the high-, medium- and low-concentration IQC samples were all under control according to the Westgard multi-rule quality control judgment rules, thereby indicating the successful establishment of the P210 IQC system. We subsequently analyzed the patient samples along with the IQC samples at the same time, and the IQC results were also under control, suggesting that the BCR-ABL P210 results of the CML patients were stable and credible (**Figures 1A–C**).

### 3.3 BCR-ABL P210 is elevated after contracting COVID-19 and is correlated with the duration of infection

**Figure 2:**
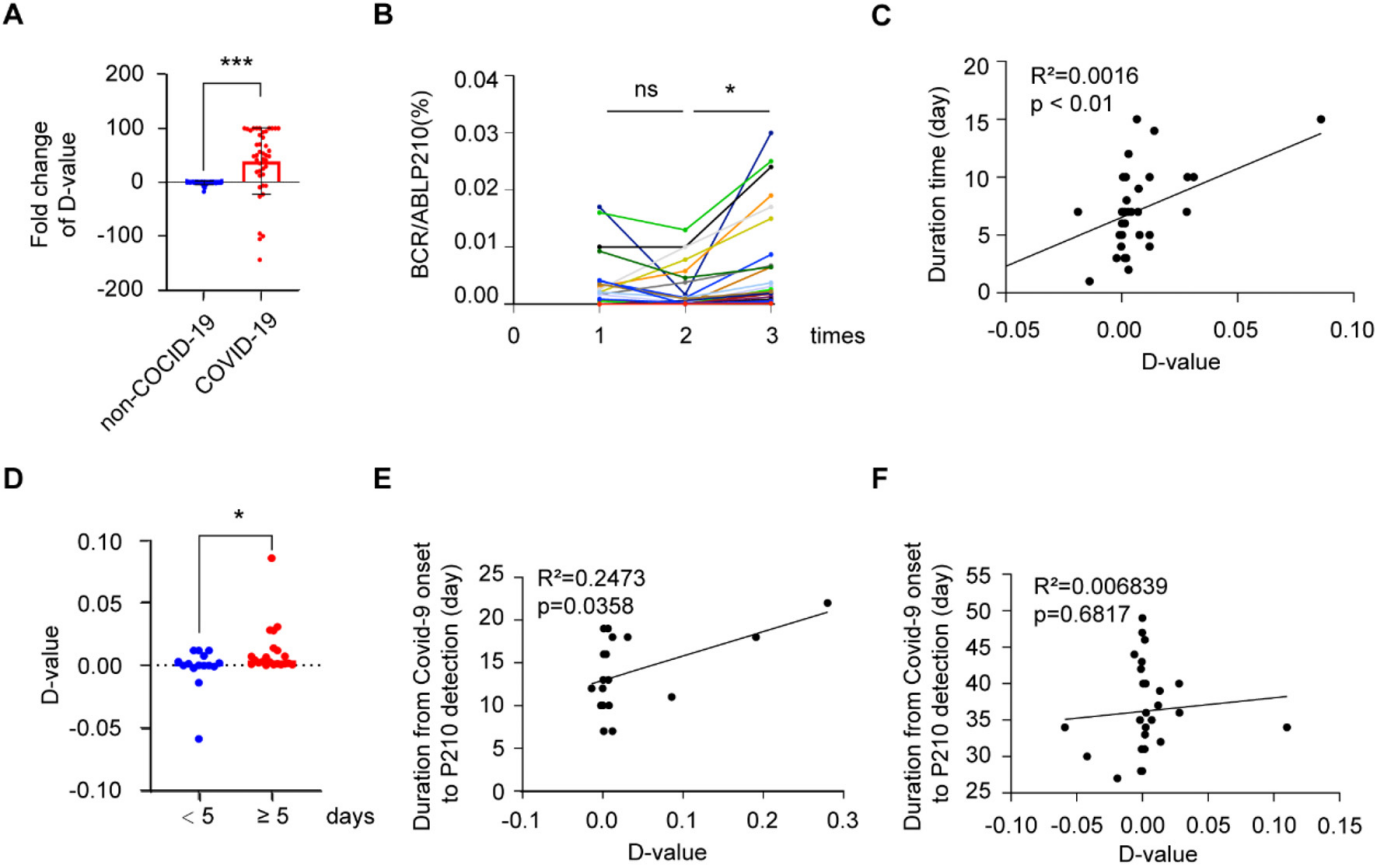
BCR-ABL P210 is elevated after COVID-19 in CML patients. **(A)** Impact of COVID-19 infection on the expression levels of BCR-ABL P210. **(B)** The change trend of BCR-ABL P210 expression level in P210^0th^, P210^1st^ and P210^2nd^. **(C)** The correlation analysis between P210 change and COVID-19 infection duration. **(D)** The change of D-value of P210 in patients of < 5 days and ≥ 5 days duration. **(E)** The correlation analysis between P210 change and duration in short-interval group (≤ 27 days). **(F)** The correlation analysis between P210 change and duration in long-interval group (> 27 days). (*p < 0.05, ***p < 0.001; ns, not significant).

We collected two blood samples, pre-infection (P210^1st^) and post-infection (P210^2nd^), from COVID-19-positive CML patients, along with two adjacent blood samples from COVID-19-negative CML patients during the same research period. We then detected their BCR-ABL P210 expression and further analyzed changes in the two sequential samples by using the difference value (D value) between the second result (P210^2nd^) and the first result (P210^1st^). We found that the number of P210-elevated patients (64.15%) in the COVID-19-infected group was significantly greater than that in the non-COVID-19-infected group (35.72%) (P = 0.003) (**Table 2**). We further analyzed the quantitative change in BCR-ABL P210 expression and found that the BCR-ABL P210 expression level was significantly elevated in the COVID-19 group compared with that in the non-COVID-19 group (P<0.001) (**Figure 2A**). At the same time, we verified the results by digital PCR for the same patients, and the results obtained were consistent with those of real-time fluorescence quantitative PCR (**Supplementary Figure 1A**). Furthermore, to exclude unknown influencing factors during the research period, we retrieved one P210 detection result before P210^1st^ detection (namely, P210^0th^) and analyzed the P210 change trend (n = 23). The results revealed no significant difference between P210^1st^ and P210^0th^ but a significant increase between P210^1st^ and P210^2nd^ (P = 0.0187) (**Figure 2B**). These results indicate that COVID-19 contributes to an increase in the BCR-ABL P210 level.

**Table 2:** Analysis of chi-square test between infected group and non-infected group in CML.

	COVID-19 ART		Total	X^2^	*P*
	negative	positive			
P210 decrease after infection	36	19	55		
P210 increase after infection	20	34	54	8.808	0.003**
Total	56	53	109		

**p< 0.01.

COVID-19, Coronavirus disease 2019; ART, Antigen rapid test.

As the duration of COVID-19 has been reported to be important for disease progression (15), we further explored the association between COVID-19 duration and P210 alteration. Our results revealed that the increase in P210 was positively correlated with the duration of COVID-19 (**Figure 2C**). When we divided the patients into two groups by a duration of 5 days, we found that the D value of P210 elevation in patients with a duration of < 5 days was significantly lower than that in patients with a duration of ≥ 5 days (P = 0.0451) (**Figure 2D**). Moreover, since the recovery time after infection might affect the P210 expression level, we further investigated the association between P210 level alteration and the interval from COVID-19 onset to detection. There was no significant correlation between P210 elevation and interval time (**Supplementary Figure 1B**). However, when we arbitrarily divided these patients into short-interval groups and long-interval groups according to the median time after recovery (27 days), the number of patients with elevated P210 in the short-interval group (≤ 27 days) was significantly greater than that in the long-interval group (> 27 days) (P = 0.026) (**Table 3**). Accordingly, a positive correlation between P210 elevation and interval time was found in the short-interval group but not in the long-interval group (**Figures 2E, F**). These findings indicate that P210 expression increases during COVID-19 and is soon restored to pre-infection levels after recovery.

**Table 3:** Analysis of chi-square test between short-interval group and long-interval group in COVID19-positive patients.

	Duration from COVID-19 onset to P210 detection (day)		Total	X^2^	*P*
	≤27	>27			
P210 decrease after infection	5	13	18		
P210 increase after infection	21	14	34	4.938	0.026*
Total	26	27	109		

*p< 0.05.

COVID-19, Coronavirus disease 2019.

### 3.4 BCR-ABL P210 is associated with patient characteristics

**Figure 3:**
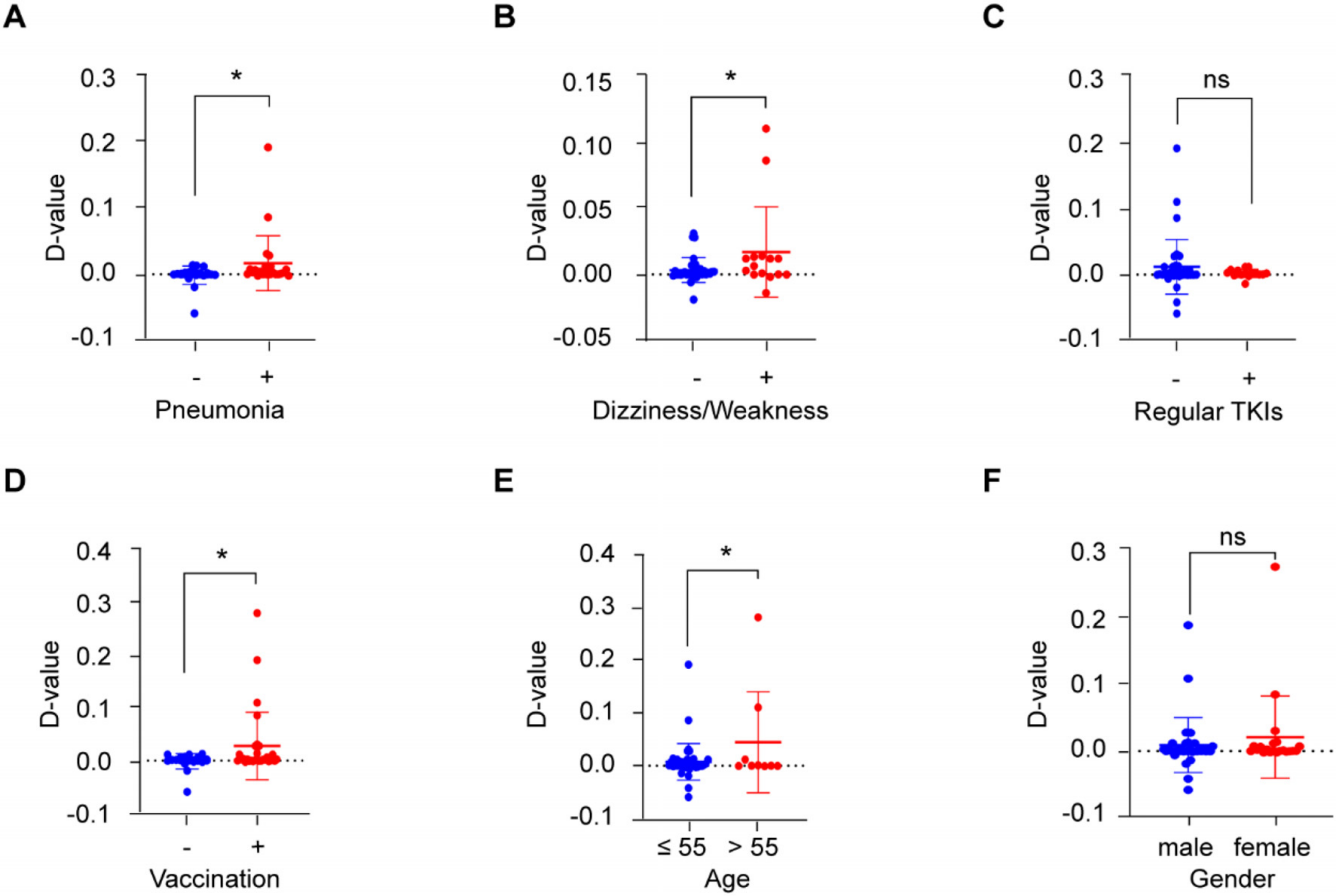
BCR-ABL P210 is correlated with COVID-19 outcomes and patients’ characteristics. **(A)** The quantitative change of BCR-ABL P210 expression when patients developed pneumonia. No pneumonia group represents with blue and pneumonia group represents with red. **(B)** The quantitative change of BCR-ABL P210 expression when patients developed dizziness and weakness. No dizziness and weakness group represents with blue and dizziness and weakness group represents with red. **(C)** The quantitative change of BCR-ABL P210 expression when patients took TKI treatment regularly. No regular TKIs group represents with blue and regular TKIs group represents with red. **(D)** The quantitative change of BCR-ABL P210 expression when patients took COVID-19 vaccination. No vaccination group represents with blue and vaccination group represents with red. **(E)** The quantitative change of BCR-ABL P210 expression when the age of patients was over 55 years. Age 55 years or less group represents with blue and over 55 years group represents with red. **(F)** The quantitative change of BCR-ABL P210 expression between female and male. Male group represents with blue and female group represents with red. (*p < 0.05; ns, not significant).

As COVID-19 patients suffer many symptoms or complications, such as cough, fever, chest discomfort, and in severe cases, respiratory distress syndrome (16), we further analyzed the correlation between BCR-ABL P210 and various symptoms of COVID-19. Our results revealed that the level of BCR-ABL P210 was significantly elevated when patients developed pneumonia or dizziness and weakness (**Figures 3A, B**), whereas there was no obvious relationship between BCR-ABL P210 and other symptoms, such as fever, nausea and emesis (**Supplementary Figures 2A, B**). In addition, taking COVID-19-treated medicines including, hormone agents and traditional Chinese medicines along with febrifuge symptom had no significant influence on the level of BCR-ABL P210 (**Supplementary Figures 2C–E**). Similarly, the use of TKI treatment regularly had no significant effect on the P210 level (**Figure 3C**). However, we found that the BCR-ABL P210 level was elevated in patients who received COVID-19 vaccination (**Figure 3D**).

Next, we further analyzed the associations of other patient characteristics with altered BCR-ABL P210 levels. We found that the level of BCR-ABL P210 was increased in patients over 55 years of age, while there was no obvious difference between females and males (**Figures 3E, F**). In addition, the counts of various blood cells, such as leukocytes, erythrocytes, platelets, lymphocytes, neutrophils, monocytes, eosinophils and basophils, were not obviously related to the BCR-ABL P210 level (**Supplementary Figures 3A–H**).

### 3.5 Dysregulated T-cell immunity is associated with an elevated BCR-ABL P210 level after contracting COVID-19

**Figure 4:**
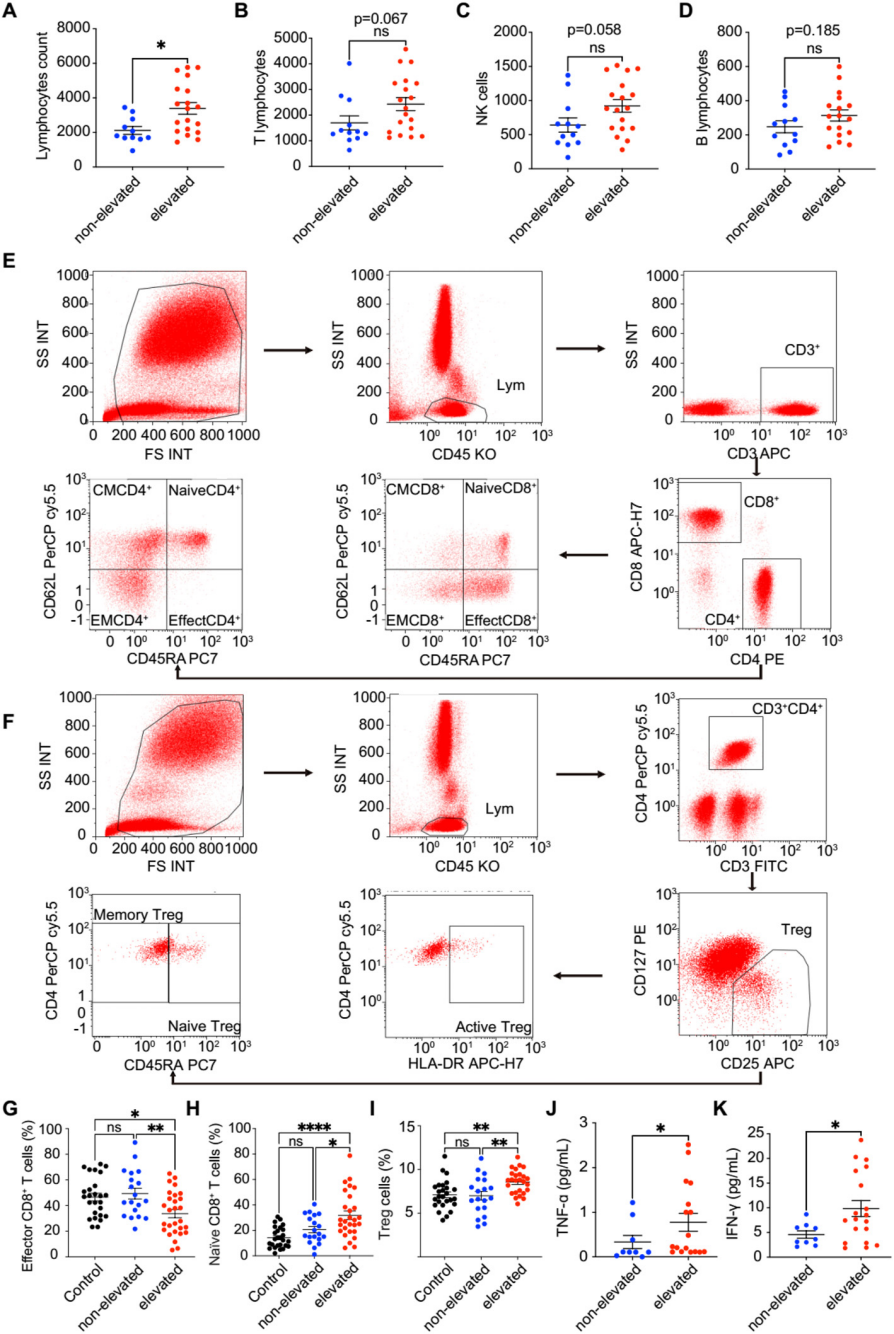
The impact of COVID-19 on lymphoid subpopulations and cytokine regulation in patients with CML. **(A–D)** The change of absolute lymphocytes, T cells, NK cells and B cells count between P210 elevated group and non-elevated group. **(E, F)** Using flow cytometry to detect for differentiating T cell subpopulations, such as CM-CD4^+^ T cells, naive CD4^+^ T cells, EM-CD4^+^ T cells, effector CD4^+^ T cells, CM-CD8^+^ T cells, naive CD8^+^ T cells, EM-CD8^+^ T cells, effector CD8^+^ T cells, naive Treg cells, memory Treg cells. **(G–I)** The change of effector CD8^+^ T cells, naive CD8^+^ T cells and Treg cells percentage between P210 elevated group and non- elevated/health control group. **(J, K)** The change of TNF-α and INF-γ in P210 elevated group and non-elevated group. (*p < 0.05; **p < 0.01; ****p<0.0001; ns, not significant).

Since immune regulation has been reported to play important roles in both COVID-19 infection and CML development, we further detected alterations in the immune cell levels of these patients. We first counted the absolute number of lymphocytes and found that P210-elevated CML patients had a greater absolute number of lymphocytes than non-P210-elevated CML patients (P = 0.012). With respect to the absolute numbers of T cells (P = 0.067), natural killer cells (NK cells) (P = 0.058) and B cells (P = 0.185), no significant difference was found; however, they had a similar trend (**Figures 4A–D**).

As T cells are important for maintaining immune homeostasis, we further analyzed subsets of T cells. Our results revealed that the percentage of effector CD8^+^ T (Teff) (CD3^+^ CD8^+^ CD45RA^+^ CD62L^-^) cells was significantly lower, whereas that of naive CD8^+^ T cells was obviously greater in P210-elevated CML patients after contracting COVID-19 than in non-P210-elevated CML patients or health controls (**Figures 4E–H**). Moreover, we found that the percentage of Treg cells was significantly increased in COVID-19-infected P210-elevated CML patients (**Figure 4I**). These findings indicate that decreased immunological function after contracting COVID-19 might contribute to an elevation in the BCR-ABL P210 level. However, there was no significant difference observed when considering other immune cells, such as central memory (CM) CD4^+^ T cells (CD3^+^ CD4^+^ CD45RA^-^ CD62L^+^), naive CD4^+^ T cells (CD3^+^ CD4^+^ CD45RA^+^ CD62L^+^), effector memory (EM) CD4^+^ T cells (CD3^+^ CD4^+^ CD45RA^-^ CD62L^-^), effector CD4^+^ T cells (CD3^+^ CD4^+^ CD45RA^+^ CD62L^-^), EM-CD8^+^ T cells (CD3^+^ CD8^+^ CD45RA^-^ CD62L^-^), CM CD8^+^ T cells (CD3^+^ CD8^+^ CD45RA^-^ CD62L^+^), naive Treg cells (CD3^+^ CD4^+^ CD127^dim+^ CD25^+^ CD45RA^+^) and memory Treg cells (CD3^+^ CD4^+^ CD127^dim+^ CD25^+^ CD45RA^-^) (**Supplementary Figures 4A–H**).

Moreover, as inflammatory cytokines are important for immunology in COVID-19 infection, we further evaluated the levels of nine cytokines via flow cytometry. The results revealed that the levels of interferon-gamma (IFN-γ) and tumor necrosis factor-alpha (TNF-α) were significantly greater in P210-elevated CML patients (**Figures 4J, K**). However, there was no significant difference in the levels of other cytokines, such as interleukin-1beta (IL-1beta), IL-2, IL-6, IL-10, IL-12P70, IL-17 and IL-4 (**Supplementary Figures 5A–G**). These results suggest that P210-elevated CML patients might have a much stronger immunological response during the process of COVID-19.

### 3.6 SARS-CoV-2 pseudovirus increases BCR-ABL P210 level *in vitro*

**Figure 5:**
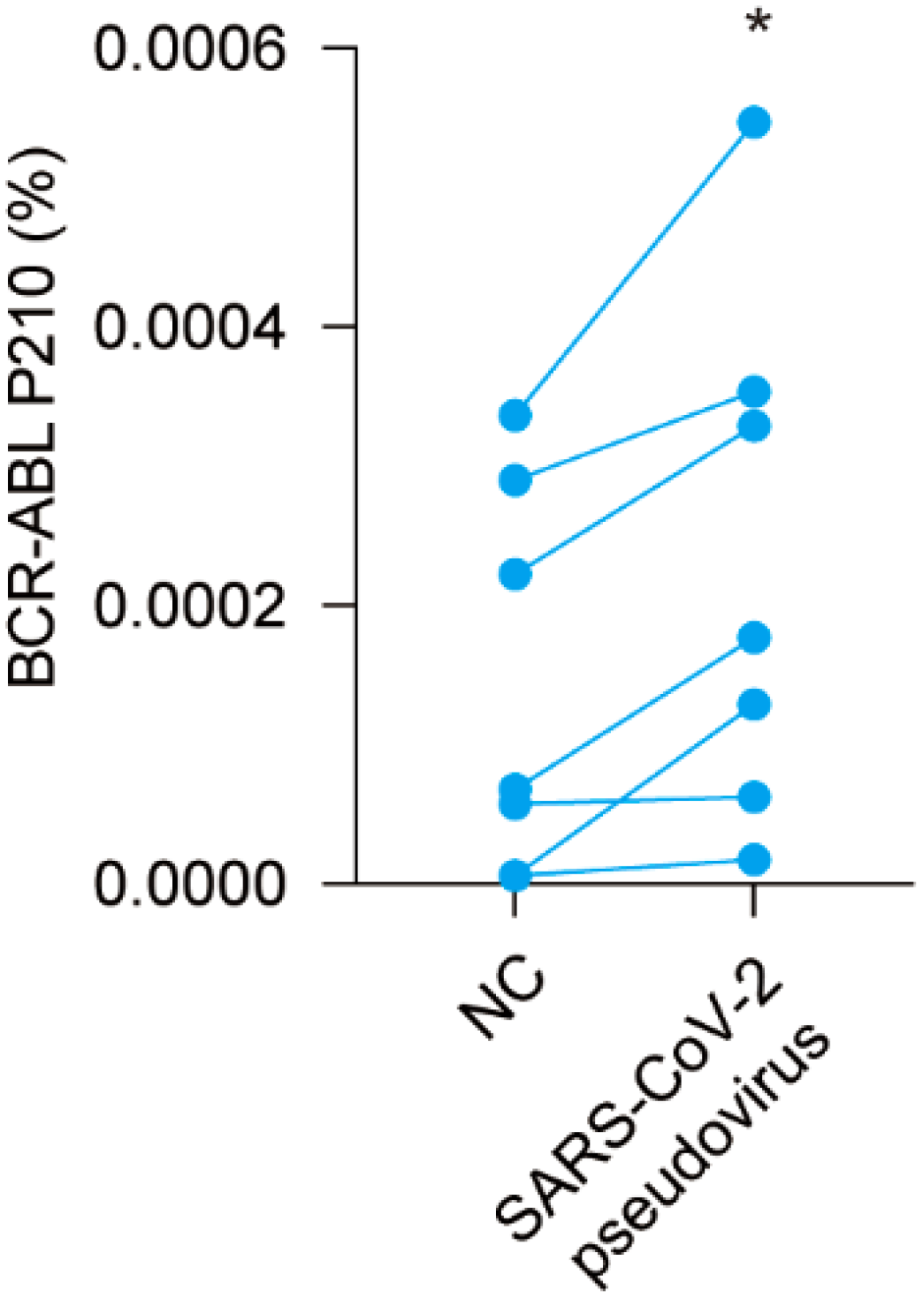
SARS-CoV-2 pseudovirus upregulates BCR-ABL P210 level *in vitro*. Pseudo-COVID-19 was added into peripheral blood mononuclear cells of CML patients to detect the difference of BCR-ABL P210 before and after adding SARS-CoV-2 pseudovirus. (*p < 0.05).

To further verify the role of COVID-19 regarding the increase in BCR-ABL P210 level, we next isolated PBMCs from CML patients and infected them with a SARS-CoV-2 pseudovirus. Subsequently, we determined alterations in the BCR-ABL P210 level. Our results revealed that the level of BCR-ABL P210 clearly increased after adding the SARS-CoV-2 pseudovirus, which was consistent with the phenomenon observed in CML patients infected with the COVID-19 (**Figure 5**). Thus, SARS-CoV-2 pseudovirus increases BCR-ABL P210 levels *in vitro*.

## 4 Discussion

In this study, we elucidated the effect of COVID-19 on the MRD level of TKI therapy and explored its immune mechanism. The level of BCR-ABL P210 in CML patients was found to be significantly elevated after contracting COVID-19. Additionally, elevated BCR-ABL P210 levels were associated with COVID-19 vaccination. We revealed that COVID-19 could inhibit immune activity by decreasing the number of effectors (CD8^+^ T cells) and increasing the number of Treg cells, which correlated with an elevated BCR-ABL P210 level. Our results indicate that COVID-19 suppresses immune activity and leads to an increase in the level of BCR-ABL P210 among CML patients.

CML is a severe hematological malignancy characterized by a Ph chromosome and BCR-ABL P210 fusion gene. However, because of the prevalence of COVID-19, CML patients, to some extent, face challenges (17). Although many studies have revealed that there are no obvious severe outcomes in CML patients contracting COVID-19, COVID-19 can trigger life-threatening complications, such as tumor lysis syndrome (TLS), which is characterized by lytic tumor cells releasing their contents into the bloodstream and jeopardizing homeostasis (10). Similarly, it has been reported that patients with hematological malignancies contracting COVID-19 are at increased risk of severe illness and have increased mortality (18). In addition, through a retrospective population-based cohort study in Canada, patients with hematological malignancies had the highest risk of severe COVID-19 and had negative COVID-19-associated outcomes when compared with patients with solid and individuals without cancer (19). Consequently, monitoring variations in CML indicators during the course of COVID-19 is highly important to avoid irreversible consequences.

In this study, we found that the level of BCR-ABL P210 was significantly elevated in CML patients after contracting COVID-19, which was also demonstrated by detecting the difference in the BCR-ABL P210 level in mononuclear cells from CML patients after infection with a SARS-CoV-2 pseudovirus. Moreover, we identified several clinical characteristics associated with an increased BCR-ABL P210 level during the process of COVID-19, such as pneumonia, drowsiness and fever. The immune response elicited by the body after vaccination is termed active immunity or acquired immunity. In this process, the immune system is activated. In addition, memory cells induced by COVID-19 vaccines play an important role in vaccine immunity (20). In this study, we found that the BCR-ABL P210 level was significantly elevated in patients who received COVID-19 vaccination.

After vaccination against COVID-19, T-cell immunity (such as the Th1 cell response), B-cell immunity (such as the germinal center response), and other immune responses may result (21, 22). Differentiated Th cells can enhance the immune response in the body by promoting the activation of CD8^+^ T cells and the secretion of IFN-γ (20). When patients suffer from COVID-19, innate inflammatory responses and adaptive immune responses become uncontrolled or impaired, which correlate with the severity of viral evasion (23). Chemokines such as INF-γ induced-protein-10 (IP-10) and immune cells such as NK cells could be predictive indicators of severe COVID-19 (24). Whether individuals with CML are immune compromised is controversial (25). Since COVID-19 and BCR-ABL P210 are tightly correlated with the immune response, we showed that T-cell immunity was involved. The number of effector CD8^+^ T (Teff) cells decreased in the BCR-ABL P210-elevated group, whereas naive CD8^+^ T cells and Treg cells increased in the same group, indicating that decreased T-cell immunity might contribute to the increase in the BCR-ABL P210 level of CML patients after contracting COVID-19.

There are several limitations in this study. It is necessary to increase the number of samples from CML patients to further verify the difference in BCR-ABL P210 levels after contracting COVID-19. In addition, the specific mechanism regulating the alterations in BCR-ABL P210 levels still requires exploration. Moreover, it is better to establish a more efficient communication web among patients, laboratories and physicians (26). Therefore, on the basis of the essential role of BCR-ABL P210 in both CML diagnosis and COVID-19, further exploration of the mechanism behind BCR-ABL P210 level alteration and its use as a bridge to monitor disease progression would be beneficial.

## Data Availability

The raw data supporting the conclusions of this article will be made available by the authors, without undue reservation.

## Supplementary material

Supplementary Figure 1 BCR-ABL P210 is elevated after contracting COVID-19 in CML patients. **(A)** Impact of COVID-19 infection on the expression levels of BCR-ABL P210 with real-time qPCR and digital PCR. The same color represents the same patient. **(B)** The association between P210 change and duration from COVID-19 onset to detection.

Supplementary Figure 2 The change of BCR-ABL P210 with COVID-19 outcomes and patients’ characteristics. **(A)** The quantitative change of BCR-ABL P210 expression when patients developed fever. Under 38.5 group represents with blue and At least 38.5 group represents with red. **(B)** The quantitative change of BCR-ABL P210 expression when patients developed nausea and emesis. No nausea and emesis group represents with blue and nausea and emesis group represents with red. **(C)** The quantitative change of BCR-ABL P210 expression when patients take hormone agents. No hormone agents group represents with blue and hormone agents group represents with red. **(D)** The quantitative change of BCR-ABL P210 expression when patients take Chinese traditional medicine. No Chinese traditional medicine group represents with blue and Chinese traditional medicine group represents with red. **(E)** The quantitative change of BCR-ABL P210 expression when patients treated with febrifuge. No febrifuge group represents with blue and febrifuge group represents with red. (ns, not significant).

Supplementary Figure 3 The relationship between the D-value of BCR-ABL P210 expression and the count of various blood cells. **(A)** The relationship between the D-value of BCR-ABL P210 expression and WBC counts. **(B)** The relationship between the D-value of BCR-ABL P210 expression and RBC counts. **(C)** The relationship between the D-value of BCR-ABL P210 expression and PLT counts. **(D)** The relationship between the D-value of BCR-ABL P210 expression and NEU counts. **(E)** The relationship between the D-value of BCR-ABL P210 expression and LYM counts. **(F)** The relationship between the D-value of BCR-ABL P210 expression and MONO counts. **(G)** The relationship between the D-value of BCR-ABL P210 expression and EOS counts. **(H)** The relationship between the D-value of BCR-ABL P210 expression and BAS counts.

Supplementary Figure 4 The impact of COVID-19 on lymphoid subpopulations in CML patients. **(A)** The change of CM-CD4^+^T cells percentage between P210 elevated group and non-elevated group. **(B)** The change of naïve CD4^+^T cells percentage between P210 elevated group and non-elevated group. **(C)** The change of EM-CD4^+^T cells percentage between P210 elevated group and non-elevated group. **(D)** The change of effector CD4^+^ T cells percentage between P210 elevated group and non-elevated group. **(E)** The change of CM-CD8^+^T cells percentage between P210 elevated group and non-elevated group. **(F)** The change of EM-CD8^+^T cells percentage between P210 elevated group and non-elevated group. **(G)** The change of naïve Treg cells percentage between P210 elevated group and non-elevated group. **(H)** The change of memory Treg cells percentage between P210 elevated group and non-elevated group. (ns, not significant).

Supplementary Figure 5
**(A–G)** The change of seven cytokines in P210 elevated group and non- elevated group, such as IL-1β, IL-2, IL-6, IL-10, IL-12P70, IL-17 and IL-4. (ns, not significant).
