# Supplementary material for: COVID-19 mitigates the response to TKIs in patients with CML via the inhibition of T-cell immunity
Source: Front Immunol. 2024 Nov 20;15:1452035. doi: 10.3389/fimmu.2024.1452035 (PMC11615079; doi:10.3389/fimmu.2024.1452035)
Supplement: Supplementary Figure 1 — BCR-ABL P210 is elevated after contracting COVID-19 in CML patients. (A) Impact of COVID-19 infection on the expression levels of BCR-ABL P210 with real-time qPCR and digital PCR. The same color represents the same patient. (B) The association between P210 change and duration from COVID-19 onset to detection. [file Image1.pdf]

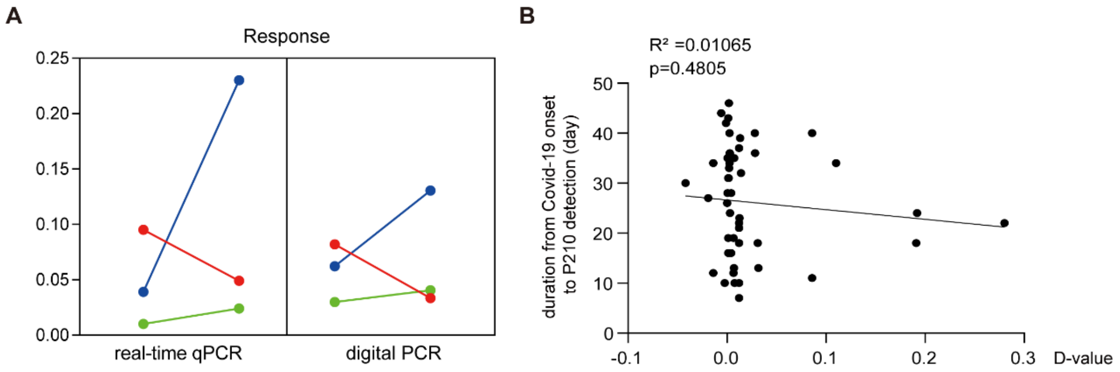

Supplementary Figure. 1 BCR-ABL P210 is elevated after contracting COVID-19 in CML patients. A Impact of COVID-19 infection on the expression levels of BCR-ABL P210 with real-time qPCR and digital PCR. The same color represents the same patient. **B** The association between P210 change and duration from COVID-19 onset to detection.
