# Supplementary material for: COVID-19 mitigates the response to TKIs in patients with CML via the inhibition of T-cell immunity
Source: Front Immunol. 2024 Nov 20;15:1452035. doi: 10.3389/fimmu.2024.1452035 (PMC11615079; doi:10.3389/fimmu.2024.1452035)
Supplement: Supplementary Figure 2 — The change of BCR-ABL P210 with COVID-19 outcomes and patients’ characteristics. (A) The quantitative change of BCR-ABL P210 expression when patients developed fever. Under 38.5 group represents with blue and At least 38.5 group represents with red. (B) The quantitative change of BCR-ABL P210 expression when patients developed nausea and emesis. No nausea and emesis group represents with blue and nausea and emesis group represents with red. (C) The quantitative change of BCR-ABL P210 expression when patients take hormone agents. No hormone agents group represents with blue and hormone agents group represents with red. (D) The quantitative change of BCR-ABL P210 expression when patients take Chinese traditional medicine. No Chinese traditional medicine group represents with blue and Chinese traditional medicine group represents with red. (E) The quantitative change of BCR-ABL P210 expression when patients treated with febrifuge. No febrifuge group represents with blue and febrifuge group represents with red. (ns, not significant). [file Image2.pdf]

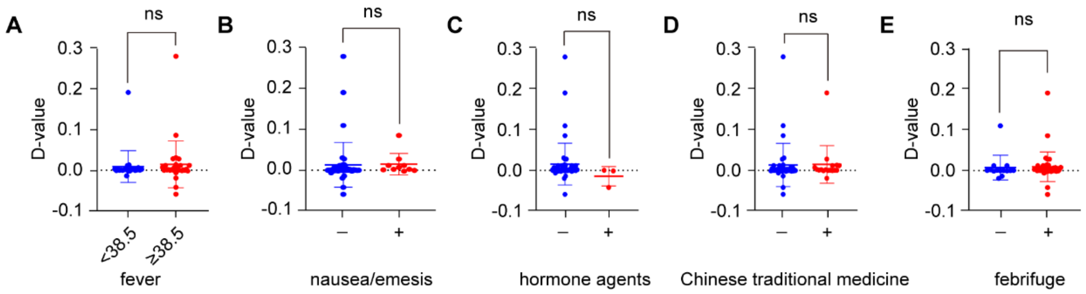

Supplementary Figure. 2 The change of BCR-ABL P210 with COVID-19 outcomes and patients' characteristics. **A** The quantitative change of BCR-ABL P210 expression when patients developed fever. Under 38.5 group represents with blue and At least 38.5 group represents with red. **B** The quantitative change of BCR-ABL P210 expression when patients developed nausea and emesis. No nausea and emesis group represents with blue and nausea and emesis group represents with red. **C** The quantitative change of BCR-ABL P210 expression when patients take hormone agents. No hormone agents group represents with blue and hormone agents group represents with red. **D** The quantitative change of BCR-ABL P210 expression when patients take Chinese traditional medicine. No Chinese traditional medicine group represents with blue and Chinese traditional medicine group represents with red. **E** The quantitative change of BCR-ABL P210 expression when patients treate with febrifuge. No febrifuge group represents with blue and febrifuge group represents with red. (ns, not significant)
