# Supplementary material for: COVID-19 mitigates the response to TKIs in patients with CML via the inhibition of T-cell immunity
Source: Front Immunol. 2024 Nov 20;15:1452035. doi: 10.3389/fimmu.2024.1452035 (PMC11615079; doi:10.3389/fimmu.2024.1452035)
Supplement: Supplementary Figure 3 — The relationship between the D-value of BCR-ABL P210 expression and the count of various blood cells. (A) The relationship between the D-value of BCR-ABL P210 expression and WBC counts. (B) The relationship between the D-value of BCR-ABL P210 expression and RBC counts. (C) The relationship between the D-value of BCR-ABL P210 expression and PLT counts. (D) The relationship between the D-value of BCR-ABL P210 expression and NEU counts. (E) The relationship between the D-value of BCR-ABL P210 expression and LYM counts. (F) The relationship between the D-value of BCR-ABL P210 expression and MONO counts. (G) The relationship between the D-value of BCR-ABL P210 expression and EOS counts. (H) The relationship between the D-value of BCR-ABL P210 expression and BAS counts. [file Image3.pdf]

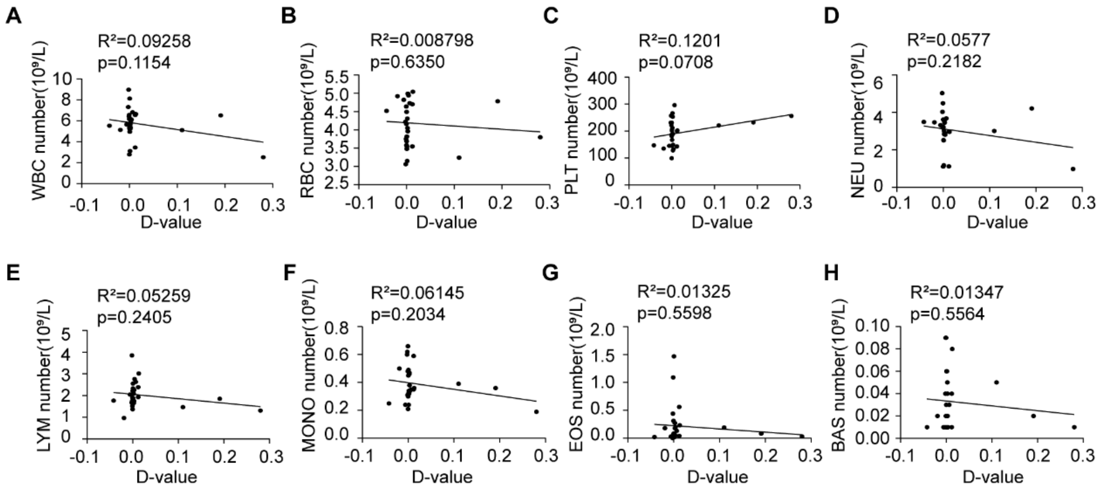

Supplementary Figure. 3 The relationship between the D-value of BCR-ABL P210 expression and the count of various blood cells. **A** The relationship between the D-value of BCR-ABL P210 expression and WBC counts. **B** The relationship between the D-value of BCR-ABL P210 expression and RBC counts. **C** The relationship between the D-value of BCR-ABL P210 expression and PLT counts. **D** The relationship between the D-value of BCR-ABL P210 expression and NEU counts. **E** The relationship between the D-value of BCR-ABL P210 expression and LYM counts. **F** The relationship between the D-value of BCR-ABL P210 expression and MONO counts. **G** The relationship between the D-value of BCR-ABL P210 expression and EOS counts. **H** The relationship between the D-value of BCR-ABL P210 expression and BAS counts.
