# Supplementary material for: COVID-19 mitigates the response to TKIs in patients with CML via the inhibition of T-cell immunity
Source: Front Immunol. 2024 Nov 20;15:1452035. doi: 10.3389/fimmu.2024.1452035 (PMC11615079; doi:10.3389/fimmu.2024.1452035)
Supplement: Supplementary Figure 4 — The impact of COVID-19 on lymphoid subpopulations in CML patients. (A) The change of CM-CD4+T cells percentage between P210 elevated group and non-elevated group. (B) The change of naïve CD4+T cells percentage between P210 elevated group and non-elevated group. (C) The change of EM-CD4+T cells percentage between P210 elevated group and non-elevated group. (D) The change of effector CD4+ T cells percentage between P210 elevated group and non-elevated group. (E) The change of CM-CD8+T cells percentage between P210 elevated group and non-elevated group. (F) The change of EM-CD8+T cells percentage between P210 elevated group and non-elevated group. (G) The change of naïve Treg cells percentage between P210 elevated group and non-elevated group. (H) The change of memory Treg cells percentage between P210 elevated group and non-elevated group. (ns, not significant). [file Image4.pdf]

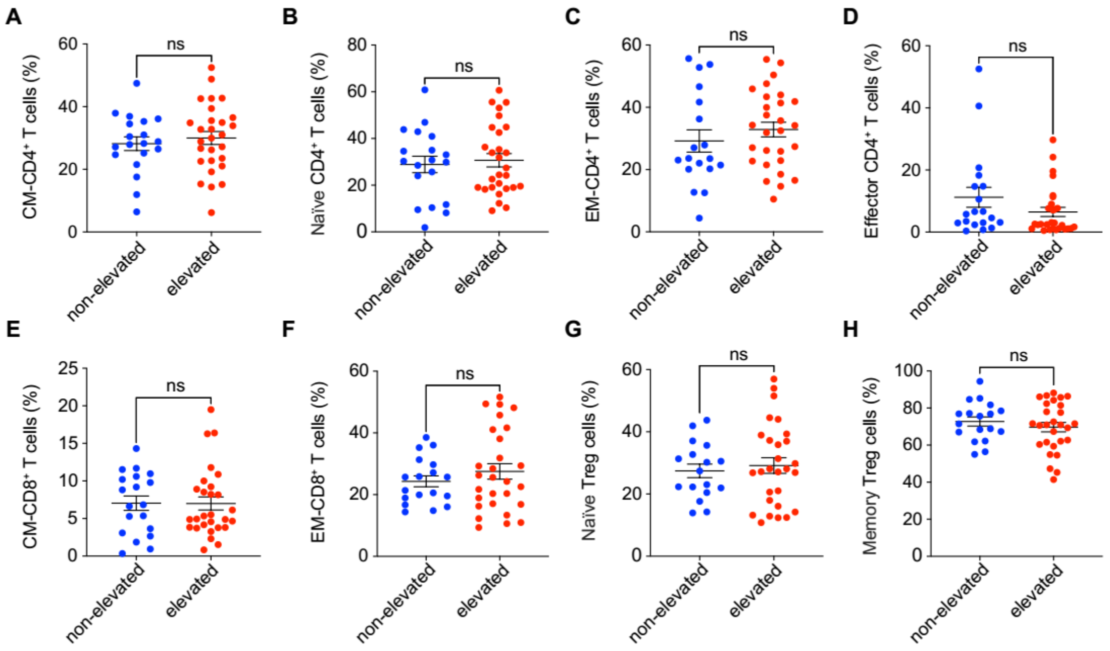

Supplementary Figure. 4 The impact of COVID-19 on lymphoid subpopulations in CML patients. **A** The change of CM-CD4$^+$T cells percentage between P210 elevated group and non-elevated group. **B** The change of naïve CD4$^+$T cells percentage between P210 elevated group and non-elevated group. **C** The change of EM-CD4$^+$T cells percentage between P210 elevated group and non-elevated group. **D** The change of effector CD4$^+$ T cells percentage between P210 elevated group and non-elevated group. **E** The change of CM-CD8$^+$T cells percentage between P210 elevated group and non-elevated group. **F** The change of EM-CD8$^+$T cells percentage between P210 elevated group and non-elevated group. **G** The change of naïve Treg cells percentage between P210 elevated group and non-elevated group. **H** The change of memory Treg cells percentage between P210 elevated group and non-elevated group. (ns, not significant)
