# Supplementary material for: COVID-19 mitigates the response to TKIs in patients with CML via the inhibition of T-cell immunity
Source: Front Immunol. 2024 Nov 20;15:1452035. doi: 10.3389/fimmu.2024.1452035 (PMC11615079; doi:10.3389/fimmu.2024.1452035)
Supplement: Supplementary Figure 5 — (A–G) The change of seven cytokines in P210 elevated group and non- elevated group, such as IL-1β, IL-2, IL-6, IL-10, IL-12P70, IL-17 and IL-4. (ns, not significant). [file Image5.pdf]

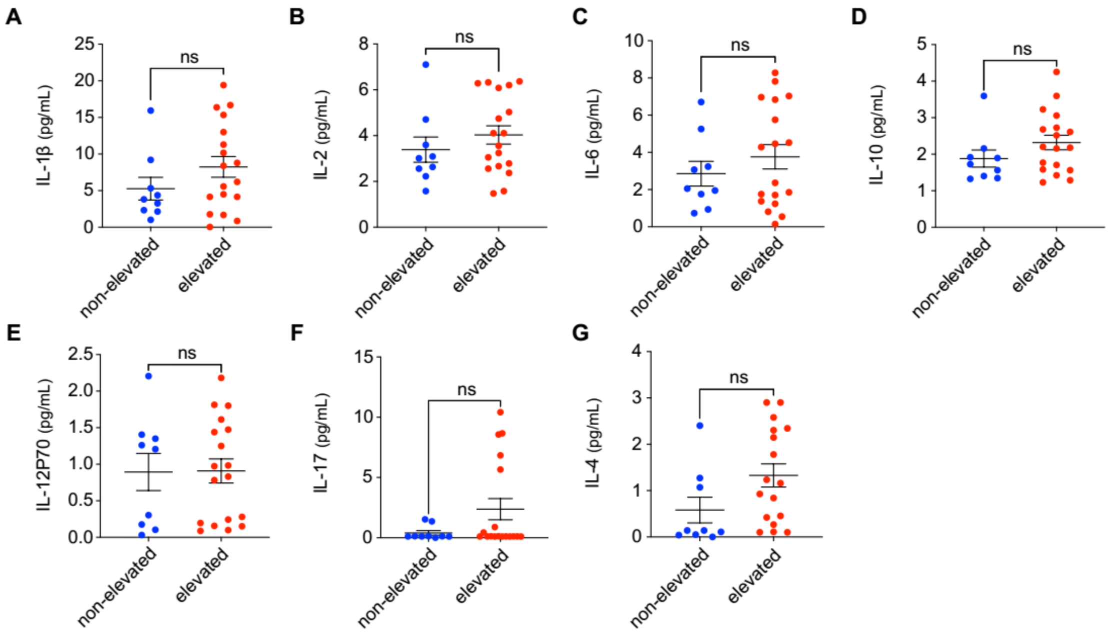

Supplementary Figure. 5 A-G The change of seven cytokines in P210 elevated group and non-elevated group, such as IL-1β, IL-2, IL-6, IL-10, IL-12P70, IL-17 and IL-4. (ns, not significant)
